# Oversleeve anastomosis in laparoscopic sphincter-preserving surgery for low rectal cancer: an overlapped end-to-end anastomosis technique without prophylactic stoma

**DOI:** 10.1093/gastro/goaa044

**Published:** 2020-08-30

**Authors:** Hao Su, Man-Dula Bao, Shou Luo, Zheng Xu, Peng Wang, Xue-Wei Wang, Chuan-Duo Zhao, Jian-Wei Liang, Qian Liu, Xi-Shan Wang, Zhi-Xiang Zhou, Hai-Tao Zhou

**Affiliations:** Department of Colorectal Surgery, National Cancer Center/National Clinical Research Center for Cancer/Cancer Hospital, Chinese Academy of Medical Science and Peking Union Medical College, Beijing, P. R. China

## Introduction

With recent advances in laparoscopic-surgery techniques and neoadjuvant therapy options, there have been improvements in sphincter-preservation outcomes in patients with low rectal cancer [1–3]. However, several issues remain controversial, such as the incidence of anastomotic leakage, the local recurrence rate, and anal-function outcome [4–5]. Moreover, prophylactic stoma at the end of sphincter-preserving surgery is necessary to prevent anastomotic leakage, especially in patients undergoing neoadjuvant chemoradiotherapy [7]. To avoid prophylactic colostomy and decrease excessive expense, we developed a new method of overlapped end-to-end anastomosis for treating low rectal cancer—a technique referred to as ‘oversleeve anastomosis.’

## The oversleeve-anastomosis procedure

Under general anesthesia, all patients were placed in the supine lithotomy position and a four-port technique was used. The operation consisted of an abdominal and a perineal phase. In the abdominal phase, the inferior mesenteric artery and vein were ligated at a high position via the middle approach. Following the total-mesorectal-excision principle, the rectum and mesorectum were dissected to the lowest point towards the pelvic floor as was possible and the pelvic autonomic nerves were protected carefully. Dissection was extended distally to expose the levator ani muscle. The intersphincteric groove was entered from the abdomen whenever possible. A hem-o-lock clip was used to mark the intestinal wall 2 cm from the distal end of the tumor. Colonic mobilization, including the release of the splenic flexure, was performed using an ultrasonic scalpel. The cutting mesenteric margin was 15 cm superior to the tumor and the cutting tract margin was 10 cm superior to the tumor. The colon was transected laparoscopically using an endoscopic linear cutter.

**Figure 1.**
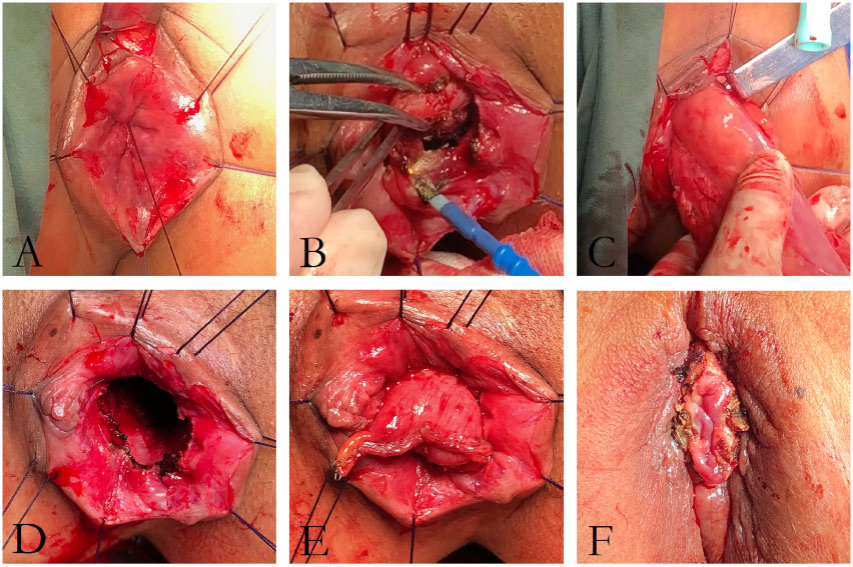
The oversleeve-anastomosis procedure in laparoscopic sphincter-preserving surgery. (A) The rectal orifice is closed; (B) circumferential incision in the rectum or anal canal; (C) the specimen is delivered through the anus; (D) the residual anorectum; (E) interrupted suture in the colon and residual rectal mucosa; (F) anastomosis ended with suture of the colon and perianal skin.

**Figure 2.**
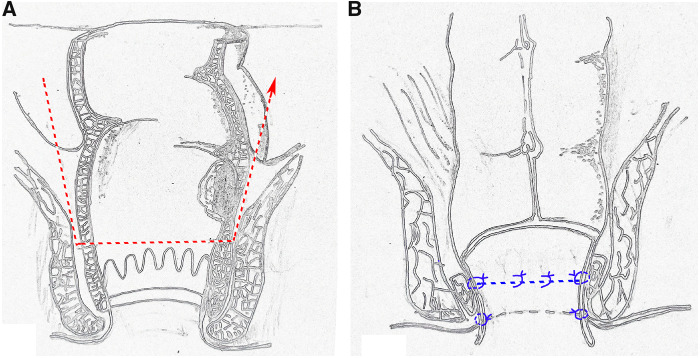
Schematic layout of oversleeve anastomosis in laparoscopic sphincter-preserving surgery. (A) Resection of the distal tract; (B) two-layer interrupted suture in oversleeve anastomosis.

The perineal phase started with finger dilatation, followed by the application of traction threads to retract the anal canal for comfortable access. The rectal orifice was then closed transanally using purse-string sutures, 1 cm below the tumor, to prevent tumor-cell dissemination during the perineal approach (Figure 1A). A circumferential incision was made in the rectum or anal canal below the purse-string suture to meet the dissection plane from the abdomen, directed by the hem-o-lock marker created previously (Figures 1B and 2A). The distal tract was then transected. A minimum distance of 1 cm distally was maintained and the external sphincter was preserved in all cases. Following the completion of dissection, the specimen was delivered through the anus and the sigmoid colon was pulled out through the anus (Figure 1C and D).

The broken end of the sigmoid colon was pulled 2 cm outside the anal verge to initiate the oversleeve anastomosis. We then performed six to eight interrupted absorbable sutures in the sigmoid colon and the residual anorectum (Figure 1E). We fixed the distal end of the colon with perianal skin using another six to eight interrupted absorbable sutures and opened the broken end of the sigmoid colon (Figures 1F and 2B). Finally, the surgery was accomplished without prophylactic stoma.

## Practical application

Between 1 April 2018 and 1 December 2018, we performed this anastomotic procedure in 21 patients with low rectal cancer, including 11 males and 10 females. Nine patients received neoadjuvant chemoradiotherapy and six received neoadjuvant chemotherapy. The mean body mass index was 23.3 kg/m² (range, 18.0–28.1 kg/m²) and the mean distance between the tumor and the anal verge by digital rectal examination was 4.1 cm (range, 3–5 cm). The mean operative time was 167.0 minutes (range, 104–300 minutes) and the mean intraoperative blood loss was 33.0 ml (range, 20–100 ml). Pathological examination showed negative distal and negative circumferential resection margins in all patients. The mean distal resection margin was 1.5 cm (range, 1.0–2.5 cm). Oral intake was started on the first post-operative morning and the mean time to first flatus was 28.9 hours (range, 15–48 hours). The mean post-operative hospitalization time was 7.7 days (range, 4–13 days) and the mean hospitalization cost was 48,114.8 CNY (range 40,022–65,664 CNY).

With a mean follow-up of 5.5 months (range, 3–11 months), the colonic stump retracted back into the rectal cavity after a mean post-operative time of 11.8 days (range, 6–21). Two patients (9.5%) experienced anastomosis leakage and were treated with transverse colostomy. None of the patients experienced symptoms indicative of anastomotic stenosis or bowel obstruction. The low anterior resection syndrome score [7] was used to evaluate anal function at the first, third, and sixth months after surgery and the results are shown in Table 1. Most patients had poor continence within 3 months after surgery. They had approximately 5–10 bowel movements per day and needed oral intake of montmorillonite powder and loperamide hydrochloride capsules to control the frequency. The anal function gradually recovered 3 months after surgery.

**Table 1.** The low anterior resection syndrome (LARS) score

Time after surgery	No LARS	Minor LARS	Major LARS
1 month, *n* (%)	1 (4.8)	5 (23.8)	15 (71.4)
3 months, *n* (%)	6 (28.6)	7 (33.3)	8 (38.1)
6 months, *n* (%)	8 (38.1)	9 (42.9)	4 (19.0)

## Discussion

An overlapped end-to-end anastomosis, a new method of digestive-tract reconstruction that we developed, looks like an oversleeve, so we named this procedure ‘oversleeve anastomosis.’ In this surgery, the specimen is extracted from the rectal stump completely without abdominal incisions, which is less invasive, better cosmetically, and decreases the incidence of incisional infection and hernia [8]. The absence of an abdominal incision leads to less post-operative pain, early recovery of gastrointestinal function, and early ground activities. Moreover, anastomotic leakage would not occur in principle, because traditional anastomosis does not exist in this surgery. Oversleeve anastomosis makes a temporary stoma unnecessary, and avoids stoma complications and the subsequent stoma-closure operation. Therefore, we safely began oral intake on the first post-operative morning, which met the requirement for enhanced recovery after surgery. All of the above are associated with short post-operative hospitalization and low hospitalization cost. Unfortunately, two patients in our study experienced anastomotic leakage on post-operative days 3 and 5, respectively; we attributed it to early retraction of the colonic stump, because we only performed interrupted sutures between the colon and residual rectal mucosa without fixation between the colon and the perianal skin, initially. Thus, we fixed the distal end of the colon both to residual rectal mucosa and the perianal skin after that, and then the two-layer interrupted sutures between the colon and anorectum ensure a practical and reliable anastomosis.

In this study, 71.4% of the patients received neoadjuvant therapy, and most of them had unsatisfactory anal function and frequent bowel movements after neoadjuvant therapy. However, patients were occasionally willing to accept the imperfect continence that may result from sphincter-preserving surgery in order to preserve their anus. Fortunately, >80% of the patients had better continence 6 months after surgery. We associated the preservation of the pelvic autonomic nerves and the entire external sphincter with favorable anal functional outcomes in this procedure [9].

In conclusion, we introduce the application of the oversleeve-anastomosis technique in laparoscopic sphincter-saving surgery for low rectal cancer. The simplicity and safety of this method might make it an ideal anastomosis technique for laparoscopic sphincter-preserving surgery.

## Authors’ contributions

H.S. collected the data and drafted the manuscript; H.-T.Z. and Z.-X.Z. designed the study and helped revise the manuscript; S.L., Z.X., P.W. and X.-W.W. collected the surgical specimens; C.-D.Z. and M.-D.B. conceived the study and participated in its coordination; J.-W.L., Q.L. and X.-S.W. participated in the data interpretation; All authors have read and approved the final manuscript.

## Funding

This work was supported by the Beijing Terry Fox Run Foundation of Cancer Foundation of China [No. LC2016B10], the Chinese Academy of Medical Sciences Initiative for Innovative Medicine [CAMS-2017-I2M-4-002], and the Postgraduate Innovation Fund Project of Peking Union Medical College in 2018 [2018-1002-02-26].
